# Knee laxity and functional knee outcome after contralateral ACLR are comparable to those after primary ACLR

**DOI:** 10.1007/s00167-020-06417-3

**Published:** 2021-01-23

**Authors:** Riccardo Cristiani, Sofia Viheriävaara, Per-Mats Janarv, Gunnar Edman, Magnus Forssblad, Anders Stålman

**Affiliations:** 1 Department of Molecular Medicine and Surgery, Stockholm Sports Trauma Research Center, Karolinska Institutet, Stockholm, Sweden; 2 Capio Artro Clinic, FIFA Medical Centre of Excellence, Sophiahemmet Hospital, Valhallavägen 91, 11486 Stockholm, Sweden; 3 Faculty of Medicine and Surgery, Karolinska Institutet, Stockholm, Sweden

**Keywords:** Anterior cruciate ligament, Primary ACL reconstruction, Contralateral ACL reconstruction, Knee laxity, KOOS

## Abstract

**Purpose:**

To evaluate and compare knee laxity and functional knee outcome between primary and contralateral anterior cruciate ligament (ACL) reconstruction.

**Methods:**

Patients who underwent primary and subsequent contralateral ACL reconstruction (ACLR) at Capio Artro Clinic, Stockholm, Sweden, from 2001 to 2017, were identified in our local database. The inclusion criteria were: the same patients who underwent primary and contralateral hamstring tendon or bone-patellar tendon-bone autograft ACLR and no associated ligament injuries. The KT-1000 arthrometer, with an anterior tibial load of 134 N, was used to evaluate knee laxity preoperatively and 6 months postoperatively. The Knee injury and Osteoarthritis Outcome Score (KOOS) was collected preoperatively and at the 1-year follow-up.

**Results:**

A total of 326 patients with isolated primary and contralateral ACLR met the inclusion criteria (47.9% males; mean age at primary ACLR 23.9 ± 9.4 years and contralateral ACLR 27.9 ± 10.1 years). The arthrometric laxity measurements were available for primary and contralateral ACLR for 226 patients. The mean preoperative and postoperative anterior tibial translation (ATT), as well as the mean ATT reduction from preoperatively to postoperatively, did not differ significantly between primary and contralateral ACLR. The KOOS was available for primary and contralateral ACLR for 256 patients. No significant differences were found preoperatively and at the 1-year follow-up between primary and contralateral ACLR for any of the five KOOS subscales.

**Conclusion:**

The findings in this study showed that anterior knee laxity and functional knee outcome after contralateral ACLR are comparable to those after primary ACLR. It is important for clinicians to counsel patients about their expectations after contralateral ACLR. This study shows that the results after contralateral ACLR in terms of knee laxity and functional knee outcome are predictable and likely to be comparable to those after primary ACLR.

**Level of evidence:**

Level III.

## Introduction

An anterior cruciate ligament (ACL) injury is a major knee trauma, especially for the young and active population. In active patients engaged in cutting and pivoting activities, as well as in patients presenting with functional instability, an ACL reconstruction (ACLR) is generally recommended [16]. Compared with a knee-healthy person, a patient with an ACL-reconstructed knee runs a greater risk of sustaining a new ACL injury in either knee [12]. Paterno et al. [11] reported that, in an active, young population who returned to pivoting activities after ACLR, 25.4% sustained a new ACL injury during the first 12 postoperative months. Seventy-five per cent of these injuries were to the contralateral knee. Many patients who suffer a contralateral ACL injury also undergo an additional ACLR. Data from the Swedish National Knee Ligament Registry [9] reported a contralateral ACLR rate of 3.8% at a 5-year follow-up after primary ACLR. Recent literature has focused on identifying potential risk factors for contralateral ACL injury and reconstruction [1, 6, 22, 23]. Some of the proposed risk factors are younger age [1, 6, 10], female gender [19] and return to a high activity level or cutting/pivoting activities [17, 20]. However, there is a lack of studies comparing the results of contralateral ACLR with those of primary ACLR. Patients undergoing contralateral ACLR need thorough counselling regarding their expectations after surgery. Studying a cohort of patients who consecutively underwent ACLR in both knees would accurately determine the results of contralateral ACLR in comparison with those of primary ACLR. These findings would be helpful for clinicians to inform and set expectations for patients undergoing contralateral ACLR.

The purpose of this study was to evaluate and compare knee laxity and functional knee outcome between primary and subsequent contralateral ACLR, within the same cohort of patients. It was hypothesised that knee laxity and functional knee outcome after contralateral ACLR would be comparable to those after primary ACLR.

## Materials and methods

This study was approved by the Regional Ethics Committee, Karolinska Institutet, (Dnr 2016/1613-31/2). Patients who underwent primary and subsequent contralateral ACLR at Capio Artro Clinic, Stockholm, Sweden, from 2001 to 2017, were identified in our local database. The inclusion criteria for this study were: the same patients who underwent primary and contralateral hamstring tendon (HT) or bone-patellar tendon-bone (BPTB) autograft ACLR and no associated ligament injuries. A total of 326 patients met the inclusion criteria. Two study cohorts were generated after applying the exclusion criteria. A first cohort, for the comparison of knee laxity, was established after excluding patients with missing KT-1000 measurements for primary or contralateral ACLR (*n* = 100). A second cohort, for the comparison of functional knee outcome, was established after excluding patients with missing Knee injury and Osteoarthritis Outcome Score (KOOS) scores for primary or contralateral ACLR (*n* = 70).

### Surgical technique and rehabilitation

All the patients were operated on using an autologous HT or BPTB technique for both primary and contralateral ACLR. Graft harvesting and fixation as well as meniscal repair techniques have been described earlier [2–4]. All the patients followed a standardised postoperative rehabilitation protocol. In the event of an isolated ACLR or an ACLR with simultaneous meniscal resection, full weight bearing and full range of motion were encouraged as tolerated. If a meniscal repair was performed, patients wore a hinged knee brace for 6 weeks. Flexion was limited from 0° to 30° for the first 2 weeks, from 0° to 60° for the third and fourth weeks and from 0° to 90° for the fifth and sixth weeks after surgery. Return to sport was allowed after 6 months at the earliest.

### Evaluation

The KT-1000 arthrometer (MEDmetric, Corp., San Diego, CA, USA), with an anterior tibial load of 134 N at 20° of knee flexion, was used to evaluate anterior knee laxity preoperatively and 6 months postoperatively for primary and contralateral ACLR. At least three measurements were made for each knee and the median value was registered. All the tests were performed by experienced physiotherapists at our outpatient clinic. The preoperative and postoperative anterior tibial translation (ATT), as well as the ATT reduction from preoperatively to postoperatively of the ACL-injured knee, were expressed in millimetres.

The functional knee outcome was evaluated using the KOOS [13–15], collected preoperatively and at the 1-year follow-up for both primary and contralateral ACLR. The KOOS is a frequently used disease-specific, patient-reported outcome for measuring functional knee outcome in patients with ACL injury and ACLR [2–4, 8]. It is divided into five subscales: Pain, Knee-related Symptoms, Activities of Daily Living (ADL), Sport and Recreation and Knee-related Quality of Life (QoL). Each subscale is scored from 0, representing “extreme knee problems”, to 100, representing “no knee problems”. It is recommended to evaluate the individual subscales independently [15].

### Statistical analysis

The computations of descriptive statistics, as well as the statistical analysis, were performed using SPSS software (Version 26.0, IBM Corp., Armonk, New York, USA). All the variables were summarised with standard descriptive statistics, such as frequency, mean and standard deviations. All the distributions were checked for severe deviations from a normal distribution. No such deviations from a normal distribution were found. Comparisons between laxity preoperatively and at the 6-month follow-up and laxity reduction from preoperatively to postoperatively for primary and contralateral ACLR were made using an analysis of variance for repeated measurements. Comparisons between KOOS subscale scores preoperatively and at the 1-year follow-up and changes from preoperatively to postoperatively for primary and contralateral ACLR were also made using an analysis of variance for repeated measurements. The significance level in all the analyses was 5% (two-tailed).

## Results

**Fig. 1:**
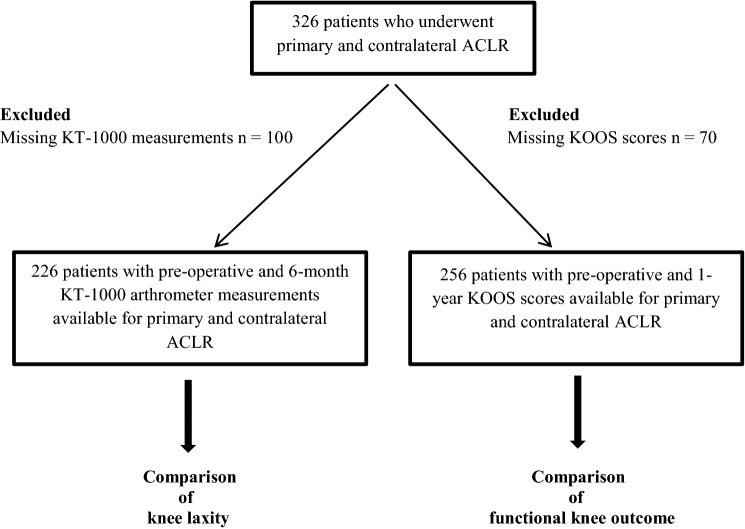
Patient flowchart. The exclusion criteria that led to the final analysis cohort groups are shown. *ACLR* anterior cruciate ligament reconstruction, *KOOS* knee injury and osteoarthritis outcome score

Patient characteristics for the entire cohort (*n* = 326) are presented in detail in Table 1. A cohort of 226 patients with preoperative and 6-month postoperative arthrometric measurements available for both surgeries was established for the comparison of anterior knee laxity between primary and contralateral ACLR. A cohort of 256 patients with preoperative and 1-year postoperative KOOS scores available for both surgeries was established for the comparison of functional knee outcome between primary and contralateral ACLR (Fig. 1).

**Table 1:** Patient characteristics

Variable		Primary ACLR	Contralateral ACLR
Gender, male/female, *n* (%)	156/170 (47.9/52.1)		
Injured side, right/left, *n* (%)	153/173 (46.9/53.1)		
Age at surgery, years ± SD		23.9 ± 9.4	27.9 ± 10.1
Activity at injury, *n* (%)			
Football		123 (37.7)	135 (41.4)
Alpine skiing		43 (13.2)	50 (15.3)
Floorball		20 (6.1)	27 (8.3)
Handball		19 (5.8)	16 (4.9)
Other sports^a^		49 (15.0)	48 (14.7)
Other		25 (7.7)	32 (9.8)
Missing		47 (14.4)	18 (5.5)
Graft, *n* (%)			
HT autograft		261 (80.1)	280 (85.9)
BPTB autograft		65 (19.9)	46 (14.1)
Associated meniscal procedures, *n* (%)			
MM resection		40 (12.3)	43 (13.2)
LM resection		45 (13.8)	54 (16.6)
MM repair		13 (4.0)	19 (5.8)
LM repair		9 (2.8)	10 (3.1)
Cartilage injuries, *n* (%)		48 (14.7)	52 (16.0)
Time intervals, months ± SD			
From injury to ACLR		10.8 ± 18.2 (*n* = 258)	10.0 ± 19.2 (*n* = 287)
From primary to contralateral ACLR	48.3 ± 39.4		

*ACLR* anterior cruciate ligament reconstruction, *BPTB* bone-patellar tendon-bone, *HT* hamstring tendons, *LM* lateral meniscus, *MM* medial meniscus, *SD* standard deviation

^a^Basketball, rugby, dancing, motocross, gymnastics, boxing, ice hockey, tennis, volleyball

### Knee laxity

**Fig. 2:**
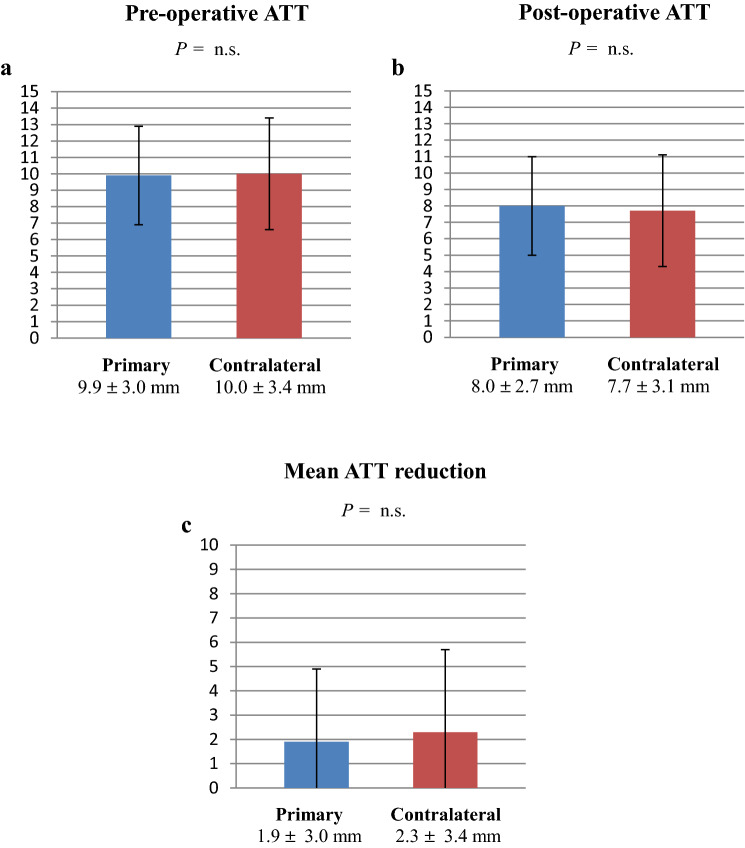
**a–c** Mean preoperative and postoperative ATT and ATT reduction measurements. *ATT* anterior tibial translation

The mean preoperative and postoperative ATT, as well as the mean ATT reduction from preoperatively to postoperatively, did not differ significantly between primary and contralateral ACLR (Fig. 2a–c).

### Functional knee outcome

**Fig. 3:**
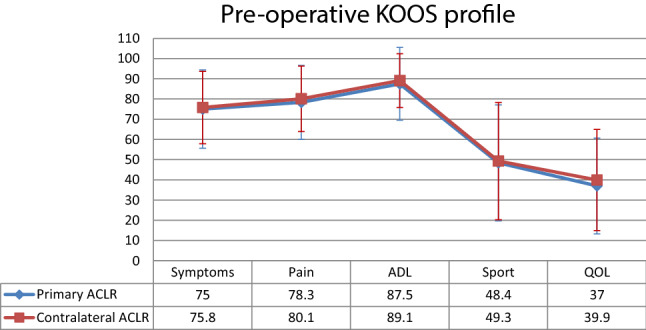
Mean preoperative scores and standard deviation per KOOS subscales for primary and contralateral ACLR. ACLR, anterior cruciate ligament reconstruction; *ADL* activities of daily living, *KOOS* knee injury and osteoarthritis outcome score, *QOL* quality of life

**Fig. 4:**
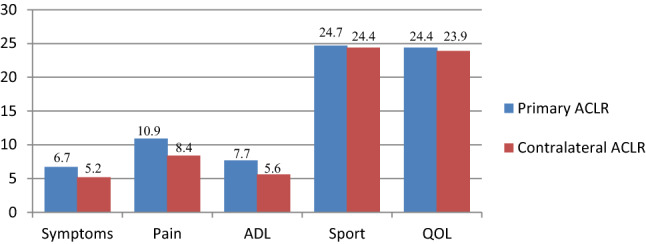
Mean improvement from preoperatively to 1-year follow-up per KOOS subscales for primary and contralateral ACLR. *ACLR* anterior cruciate ligament reconstruction, *ADL* activities of daily living, *KOOS* knee injury and osteoarthritis outcome score, *QOL* quality of life

**Fig. 5:**
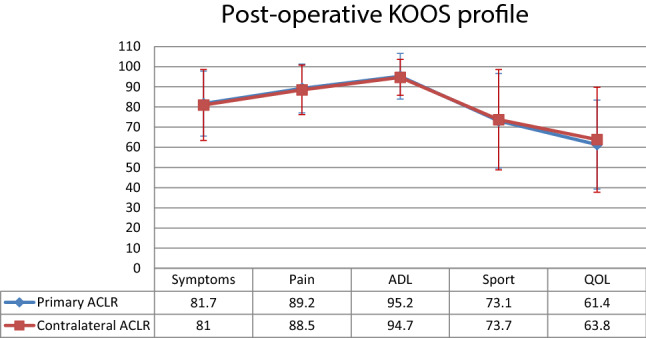
Mean postoperative scores and standard deviation per KOOS subscales for primary and contralateral ACLR. *ACLR* anterior cruciate ligament reconstruction, *ADL* activities of daily living, *KOOS* knee injury and osteoarthritis outcome score, *QOL* quality of life

The preoperative and postoperative KOOS scores, as well as the mean improvement from pre-operatively to the 1-year follow-up, did not show any significant difference between primary and contralateral ACLR for any of the five KOOS subscales (Figs. 3, 4, 5).

## Discussion

The most important finding in this study was that anterior knee laxity and functional knee outcome after contralateral ACLR were comparable to those after primary ACLR, in the same cohort of patients.

A tear of the ACL graft or a contralateral ACL tear is a devastating event for patients, often requiring surgery and repeating the long rehabilitation process. It has been estimated that there is a 3% risk of tearing the ACL graft and a similar risk of 3% for tearing the contralateral ACL during the first 2 years after ACLR [22]. However, the contralateral knee might be at greater risk than the ipsilateral knee with continued follow-up. In their systematic review, Wright et al. [23] found that, after a minimum of 5 years of follow-up, the risk of an ACL tear in the contralateral knee (11.8%) is approximately twice as high as the risk of an ACL graft rupture in the ipsilateral knee (5.8%). With regard to the contralateral ACL, most of the current literature has focused on understanding the risk factors for contralateral ACL injuries and ACLR [1, 6, 10, 17, 19, 20, 22, 23]. To date, however, there is a lack of literature comparing the results of contralateral ACLR with those of primary ACLR.

Fältström et al. [5] showed that patients with bilateral ACL injuries reported poorer knee function and quality of life compared with patients who underwent unilateral ACLR. In detail, patients with bilateral ACL injuries reported significantly lower scores on the Pain, Sport/Recreation and Quality of Life KOOS subscales in comparison with patients with a unilateral ACLR. However, their study group was smaller and more heterogeneous. Some patients had graft ruptures and revisions. In addition, all the patients with bilateral ACL injuries were included, irrespective of whether or not they underwent reconstruction. The included patients underwent surgery at different orthopaedic clinics, with different surgical methods and following different rehabilitation protocols. Finally, the study was based on a matched group analysis comparing patients with bilateral ACL injuries with patients with a unilateral ACLR.

To our knowledge, this is the first study to compare the results between primary and contralateral ACLR in the same cohort of patients. In a recent study, Cristiani et al. [2], in a similar fashion, compared the results between primary and revision ACLR in the same cohort of patients. The authors showed that revision ACLR restores knee laxity but shows inferior functional knee outcome compared with primary ACLR. There are several potential reasons for having a poorer postoperative functional knee outcome after revision ACLR in comparison with primary or contralateral ACLR. First, the patients may have more cartilage and meniscal injuries, as well as increased knee pain, due to the repeated surgical trauma after revision ACLR. Moreover, they have sustained two serious knee injuries (ACL tear and ACL graft rupture) and multiple graft harvesting of both the flexor and extensor knee mechanism [2, 7, 21]. The results of this study suggest that having a contralateral ACLR is a less dramatic event than having a revision ACLR.

With regard to the patient characteristics of our study cohort, it is interesting to note that the male/female ratio was 47.9/52.1 and the mean age at primary ACLR was 23.9 ± 9.4 years. This supports the idea that younger age [1, 6, 10] and female gender [19] may be factors associated with a higher risk of contralateral ACL injury and reconstruction. In a previous study based on data from the Swedish National Knee Ligament Registry, Kvist et al. [9] reported a male/female ratio of 58/42 for all patients who underwent primary ACLR and a mean age at primary ACLR of 28 ± 9 and 26 ± 11 years for males and females, respectively. In addition, in the present study, the most common activities in conjunction with a contralateral ACL injury were football (41.4%), alpine skiing (15.3%), floorball (8.3%) and handball (4.9%), underlining the high risk of contralateral ACL injury and reconstruction when returning to cutting/pivoting activities [17, 20].

The main strength of this study was that the results of primary and contralateral ACLR were compared directly in the same cohort of patients. This sequence of events reflects what happens in the real clinical setting. The study group was relatively large (226 patients for the comparison of knee laxity and 256 patients for the comparison of functional knee outcome) and homogeneous (all patients underwent first primary and then contralateral ACLR). Finally, all the patients underwent surgery and the preoperative and postoperative assessment for both surgeries at the same institution and the rehabilitation was standardised.

The most important limitation was that no data were available to compare the return to sport after primary and contralateral ACLR. Another limitation was the short-term follow-up. However, in a study based on the Swedish National Knee Ligament Registry, Samuelsson et al. [18] reported equivalent results for patients on KOOS subscale scores at 1- and 2-year follow-ups after ACLR, implying that there is no additional value in capturing 2-year KOOS subscale scores.

Patients undergoing contralateral ACLR need appropriate counselling regarding their expectations after surgery. Studying the same cohort of patients undergoing first primary ACLR and then contralateral ACLR makes it possible to compare the outcome of the second surgery with that of the first surgery, as this sequence of events mimics what happens in the real clinical scenario. Our findings show that the results after contralateral ACLR in terms of knee laxity and functional knee outcome are predictable and likely to be comparable with those after primary ACLR.

## Conclusion

The findings in this study showed that anterior knee laxity and functional knee outcome after contralateral ACLR are comparable to those after primary ACLR. It is important for clinicians to counsel patients about their expectations after contralateral ACLR. This study shows that the results after contralateral ACLR, in terms of knee laxity and functional knee outcome, are predictable and likely to be comparable to those after primary ACLR.
